# Evaluating the Efficacy of Case-Based Learning for Medical Oncology Trainees: A Systematic Review

**DOI:** 10.1007/s13187-025-02651-w

**Published:** 2025-06-05

**Authors:** Jonathon Vundum, Fiona Day

**Affiliations:** 1 Department of Medical Oncology, Calvary Mater Newcastle, Newcastle, NSW Australia; 2 School of Medicine and Public Health, University of Newcastle, Newcastle, NSW Australia

**Keywords:** Post-graduate, Medical oncology, Case-based learning, Problem-based learning

## Abstract

Active learning methodologies, such as case-based learning (CBL), are widely used in undergraduate medical education. CBL is associated with high student satisfaction, knowledge acquisition and the development of critical thinking abilities. The aim of this review is to determine whether these findings are generalisable to postgraduate medical oncology trainees. A systematic review was conducted using Medline and Embase, following the Preferred Reporting Items for Systematic Reviews and Meta-analyses (PRISMA) reporting guidelines. Studies were evaluated using the Critical Appraisal Skills Programme (CASP) checklist and a narrative synthesis without meta-analysis was completed to summarise findings. There is a lack of published data in this area. Three studies were eligible for review: one randomised controlled trial (RCT), one qualitative study and one educational pilot. Two studies were based in the UK and one study was based in China. In total, there were 111 participants and studies were published between 2012 and 2024. Studies universally demonstrated CBL was viewed positively by practicing doctors. Further findings included CBL being associated with perceived increases in confidence, learning and examination results. There were no reported negative aspects of CBL. All three studies had significant methodological flaws limiting the validity and reliability of stated findings. There is a lack of robust data in this field; however, the available evidence supports findings from the undergraduate setting, namely CBL is valued by trainees. Further studies are required to demonstrate whether CBL courses are feasible within speciality training programmes and whether they benefit trainee performance.

**Supplementary Information:**

The online version contains supplementary material available at https://doi.org/10.1007/s13187-025-02651-w.

## Introduction

Active learning methodologies, such as case-based learning (CBL), are employed by medical schools internationally. In the undergraduate setting, there is evidence to suggest they are more effective than traditional lecture-based learning (LBL) methods and are associated with high student satisfaction. Benefits include acquisition of knowledge, improvements in social and communication skills, problem-solving and the development of self-directed learning techniques [1, 2]. Postgraduate training has several relevant differences which may mean findings at undergraduate level are not generalisable. These include, but are not limited to, smaller trainee populations with longer placement durations and exposure to greater numbers of real patient cases. Furthermore, the focus of postgraduate training is different. There is greater emphasis on developing skills to improve physicians’ competence and performance, rather than simply increasing knowledge [3].

With regard to speciality training in medical oncology, The Royal Australian College of Physicians (RACP) mandates that medical oncology advanced trainees be exposed to a mix of cases through conducting a minimum of three outpatient clinics a week [4]. Dale’s “Cone of Experience” is widely published and provides support for these requirements. It suggests that trainees are likely to retain more knowledge through the practice of real-life oncology cases than by simply discussing them [5]. In addition to the above, RACP mandates that speciality trainees attend multidisciplinary team meetings (MDTs) [4]. These give trainees the opportunity to observe dialogs between specialists on complex real-life cases and occasionally trainees can present and discuss patients they have directly managed. These opportunities are too resource intensive to provide to all medical students and thus CBL becomes an attractive alternative at the undergraduate level.

In modern healthcare, barriers exist to optimal learning conditions in both clinics and MDTs. Outpatient appointments are aimed at providing patients with optimal care. Trainee learning is an opportune by-product of these encounters. Patient volumes and resulting time constraints limit the learning opportunities available from each patient encounter [6]. Furthermore supervising specialists vary in their availability and willingness to facilitate discussions to consolidate learning during or after clinics. Similar issues have been identified with MDTs and this can detract from trainees’ satisfaction with these learning methods, as well as their perceived ability to achieve learning goals [7, 8]. When patient care is the primary focus, trainees often only have time to consider information that is relevant to a decision at a snapshot in that patient’s cancer journey. This allows for only superficial consideration of prior patient issues, investigations and lines of therapy. Formal case-based discussions on the other hand may allow detailed exploration of patient cases from time of diagnosis to discharge or death.

## Aims

The specific aim of this review is to identify and appraise available literature examining the efficacy of CBL in populations that closely resemble that of speciality trainees in medical oncology. Our objective is to establish whether the evidence from undergraduate studies, indicating that CBL is associated with knowledge acquisition, improved communication and social skills, critical reasoning and trainee satisfaction is also applicable in a postgraduate setting.

## Methods

**Fig. 1:**
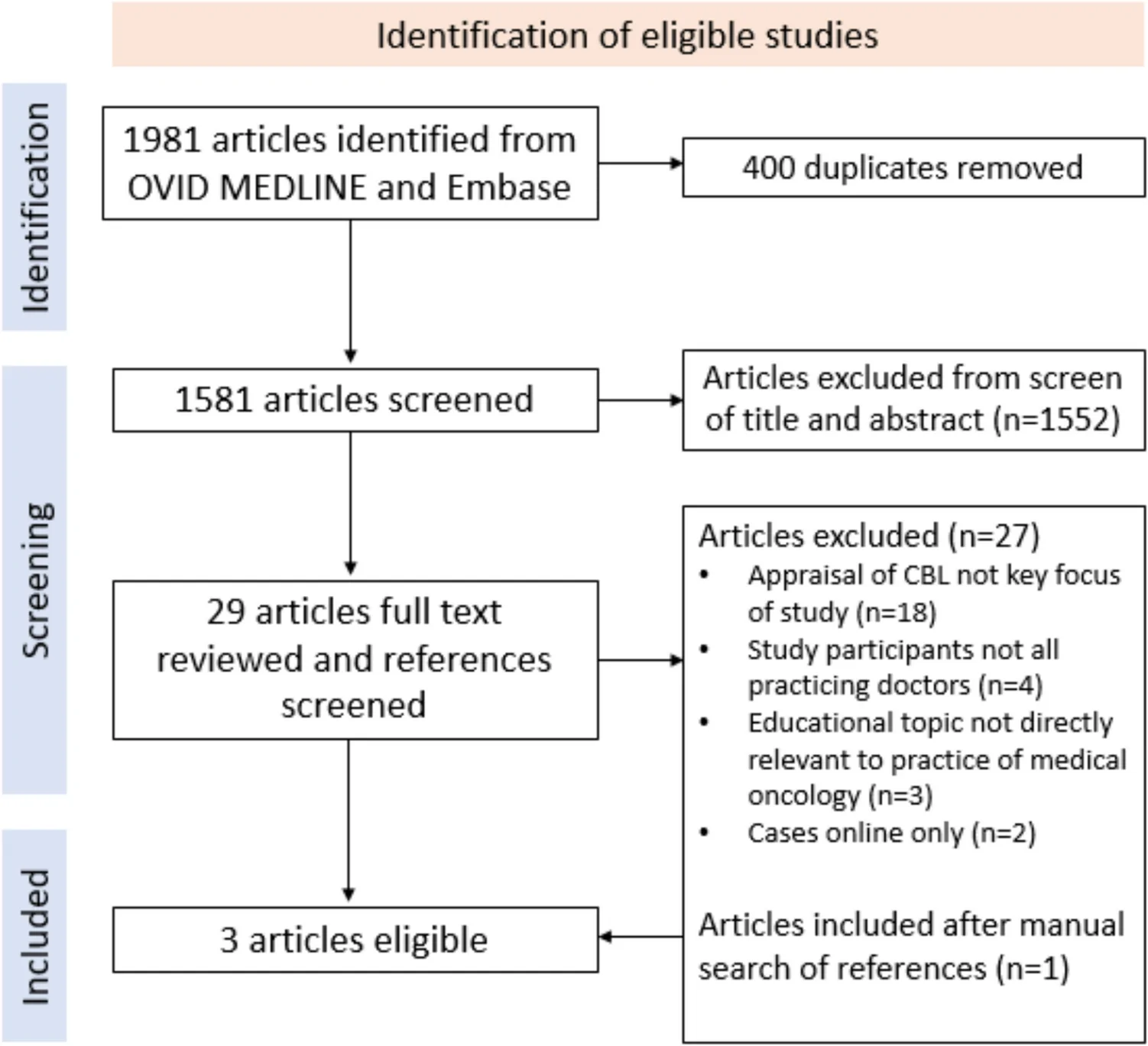
PRISMA flow diagram

This review followed the Preferred Reporting Items for Systematic Reviews and Meta-analyses (PRISMA) reporting guidelines [9] (Fig. 1). The Cochrane Handbook for Systematic Reviews of Interventions was used as a secondary reference [10].

## Eligibility Criteria

Eligible studies included those whereby: (1) appraisal of an aspect of CBL as a means of education was a key focus of the study, (2) participants were all practicing doctors and (3) the topic of learning was directly relevant to the practice of medical oncology. More specifically, papers deemed relevant to the practice of medical oncology were those focussed on cancer sciences and/or anti-cancer systemic therapies. All types of studies with full-text available were included. Exclusion criteria consisted of (1) studies primarily focused on haematological malignancies; (2) studies focussed on a local cancer therapy, such as a surgical or radiotherapy technique—that is, those more relevant to a specialty other than medical oncology; (3) studies whereby there was no requirement for face-to-face discussion of cases; and (4) publications in a language other than English.

## Search Strategy

To ensure appropriate Medical Subject Headings (MeSH) headings, a scoping search was performed on TRIP Pro using keywords [11]. The MeSH headings of relevant articles were noted and expanded in discussion with a NSW Health librarian. Searches were conducted using Medline and Embase. In addition, the reference lists of all papers that underwent full-text review (see below) were manually screened for further relevant articles. Time restriction was applied from 1996 to 3 July 2024. The complete search strategy can be found in the Supplementary Appendix.

## Selection Process

A literature search was performed and any duplicates were removed. Reviewer (JV) reviewed the titles and abstracts of all articles. Those clearly not meeting study eligibility were removed. The full text of all remaining articles was subsequently reviewed and their references were screened for further potentially eligible articles. If the articles were ineligible at this stage, the reason was documented. When there was uncertainty as to whether an article met eligibility criteria, the article was discussed with a second reviewer (FD) in order to come to a consensus.

## Data Processing

Data collected from each eligible article included details of publication (year, author, journal and country), study design, number of participants, comparison group details if applicable, outcomes, relevant results and conclusions. All data collected was uploaded to a single spreadsheet in Microsoft Excel.

In view of the small number of eligible studies and their nature, the researchers determined it best to proceed with narrative synthesis only. Reviewer (JV) assessed the eligible studies using the Critical Appraisal Skills Programme (CASP) checklist for randomised controlled trials and/or qualitative studies as appropriate considering the study’s methodology [12]. As there is no specific checklist for pilot studies, evaluation was undertaken using steps from the other checklists as deemed relevant.

## Results

The literature search identified 1981 articles. A total of 400 duplicates were identified and removed. After screening titles and abstracts of the remaining 1581 articles, a further 1552 were removed due to failure to meet eligibility criteria. Full-text reviews were undertaken for the remaining 29 articles, of which 2 were found to be eligible [13, 14]. Of the 27 publications deemed ineligible at this stage, the primary reasons were as follows: appraisal of CBL methodology did not form a key focus of the study (18); study participants were not all practicing doctors (4); the educational topic was not directly relevant to the practice of medical oncology (3); and there was no face-to-face discussion of cases (2). After a manual search of references, one article was found to be eligible [15]. One study required discussion with the second reviewer (FD) [14]. The concern was that it was not clear whether the postgraduate students in the study had completed an undergraduate medical degree. The authors of the paper and Bengbu University were contacted via email without response. Subsequently, research was conducted into the medical education pathway in China. Eligibility for postgraduate clinical speciality training in China is regulated by the National Medical Licencing Law of the People’s Republic of China, which ensures that postgraduate students in fields such as oncology require a bachelor’s degree in medicine and to have passed the National Medical Licensing Examination [16]. For this reason, the study was deemed eligible.

## Study Characteristics

The three eligible studies by M. Bi et al., R. Jyothirmayi and E. C. Mackay et al. varied in their design; they consisted of a randomised controlled trial, a qualitative study and an educational pilot study respectively. All were published between 2012 and 2024. All studies had small numbers of participants (range 12–80, total 111) and were conducted at single institutions. M. Bi et al. conducted their trial in China. The remaining two studies were conducted in the UK. Via analysis with CASP checklists, all studies were found to have serious methodological flaws that reduced the validity and reliability of their respective conclusions (Table 1).

**Table 1:** Characteristics of included studies

Author	Participants	Study design	Findings
Bi et al	80 1 st year postgraduate trainees	Randomised controlled trial	CBL was enjoyable and associated with perceived improvement in critical thinking abilities. Post test scores were significantly higher with CBL when compared to traditional teaching methods
Jyothirmayi	12 speciality registrars/consultants	Educational pilot study	Case-based discussions (CBDs) perceived as useful for teaching and learning. There was evidence of reasoning and learning occurring during CBDs
Mackay et al	19 speciality registrars	Qualitative	Trainees rated CBL as highly useful and perceived that it increased confidence and would benefit future practice

## Study Appraisal and Narrative Synthesis

### M. Bi et al. (2019) [14]

The Chinese study has a clearly formulated research question in a defined group of students and hypothesised that active learning methodologies like CBL would be more effective than traditional LBL approaches. Students were randomly assigned to an experimental group or a control group, using a random number generator at a 1:1 ratio, with the purpose of learning the program’s lung cancer curriculum. The experimental group was divided into small groups and were assigned a real patient from whom to obtain a medical history via telephone. Subsequently, in their small groups and with a teacher, the students discussed the case and identified learning goals. PowerPoint was used to summarise materials of the course. The control group received 6 sessions, lasting around 40 min each, whereby a teacher used PowerPoint to facilitate the delivery of the course curriculum and homework was assigned after each class. The number and length of sessions for the experimental group and attendance records in both groups for individual sessions are not documented. The resources required to run each arm are unclear. The authors note that participants were not informed that there was an alternative format for learning. How patients were consented for the study and whether participants remained unaware of the alternate arm for the duration of the study is unclear.

All 80 eligible participants successfully completed a dichotomous questionnaire and examination two weeks after the course finale without dropout. The authors reported statistically significant improvements in the experimental group with respect to participant satisfaction and examination results. Confidence intervals were not provided for analysis of questionnaire results and crossed the null value with respect to examination results.

Although all participants were 1 st year postgraduate oncology students at the same institution there were no further stratification factors for randomisation beyond this. The authors advise that there was no statistical difference between the two groups in terms of gender, age, entrance achievement, self-study ability and subject preference. It is unclear how self-study ability was determined. The specific statistical tests and significance level used to generate this statement were not documented. Forty per cent of the participants in the control group were male compared with 30% in the experimental group. The authors stated potential confounding variables including setting and time difference were controlled but no further evidence is offered.

The randomised control design and the attempt at blinding participants in this trial are its key strengths. Its success in blinding and thus impact on performance bias is however unclear. The key weaknesses include concerns regarding internal validity. As a result of the experimental group also receiving PowerPoint teaching to summarise learning materials, it could be argued that the study is actually examining whether CBL *and* traditional learning methodologies are better than traditional learning methodologies alone. This was not the stated aim of the study. Methodological information provided was lacking in transparency and clarity and thus the extent of other confounding factors is unclear. The use of a dichotomous questionnaire is likely overly simplistic. A linear scale would have given more insight into magnitude of effect. Although there was a trend towards improved results in the experimental group, confidence intervals were large indicating that the study was underpowered.

### R. Jyothirmayi (2012) [15]

This small qualitative study has a clear statement of its aims, specifically to determine whether case-based discussions (CBDs) can function as a teaching aid in medical education rather than existing as a mere assessment tool. The study included 12 participants: 6 clinical oncology speciality registrars and 6 consultant clinical oncologists. Its goal was to assess the perceptions of teachers and learners in relation to the benefits of CBDs. This was achieved by recording and transcribing CBDs and subsequently conducting semi-structured interviews with trainees. In view of the study’s stated aims, its qualitative research methodology was appropriate. Another aim of the study was to determine the role of CBDs in teaching and learning.

The recruitment strategy employed in this study is unclear. Specifically, there are no documented inclusion or exclusion criteria and it is uncertain whether any invited consultants or trainees declined participation. In addition, of the six trainees included in the study, only three were involved in CBDs that were transcribed and the exact number of CBDs that each of these three trainees contributed to was not documented. The researcher was a consultant in the same department as the trainees and conducted four out of the six CBDs. It is not clear whether any of the extracts from CBD transcripts that were documented in the paper as evidence of learning and teaching were from discussions that did not involve the primary researcher.

Transcripts of CBDs were recorded to allow conversational analysis which was used to identify *features indicative* of teaching and learning. The author acknowledged that the study did not examine knowledge retention after these sessions. Analysis of themes identified during interviews was performed solely by the researcher and all themes generated were not documented. Semi-structured interviews with trainees were conducted by the researcher. The study concluded that reasoning and learning were taking place during CBDs and that they were perceived as useful.

A strength of this study is that it increases awareness with regard to the potential educational opportunities of CBDs. This is relevant to trainees in Australia given the RACP mandates at least two CBDs each year [17]. The author recognises that the small sample size and susceptibility to bias limit the validity of the study. The extent to which this is the case is understated. Conclusions are potentially significantly affected by researcher, selection and conformational bias severely limiting ability to draw valid conclusions from this study.

### E. C. Mackay et al. (2024) [13]

This educational pilot study had the clear stated aims of (1) assessing whether a simulated cancer multi-disciplinary team (MDT) meeting could be effective in improving trainee confidence in subsequent MDTs and (2) if it would lead to a change in trainee practice and prepare them for leadership roles. It included 11 oncology specialist registrars and 8 respiratory specialist registrars. Trainees attended a 3-h interactive CBL session. This included discussion of four patient scenarios whereby the cases were designed to mirror the complexities of real-life oncology patients. Trainees completed pre- and post-course questionnaires in order to assess perceptions of the session’s utility and whether it improved trainee confidence and altered future clinical practice.

All participants completed the questionnaire without dropout. The relationship between the researcher and the participant trainees was unclear and questionnaires were not completed anonymously. Improvement in trainee confidence was found to be statistically significant (*p* < 0.01) using a paired t test. The magnitude of effect, an increase of 2 on a 10-point scale, was a meaningful increase. No confidence intervals were generated and as such it was unclear where the true mean difference existed. Qualitative data was generated and evidenced that a significant number of trainees valued interdisciplinary sessions.

The study’s strengths include its identification of an area of need with regard to trainee dissatisfaction with current training around MDT participation. Considering its pilot design, its endpoints are overly ambitious and discordant. Considering Bowen’s key areas of focus for feasibility [18], the study’s conclusions could have been more valid by considering the extent to which trainees believe further simulated sessions would help them achieve their learning goals and whether trainees believe the resources (particularly time) would be available to enable further sessions. To truly establish the stated aims of this project, a prospective cohort approach would be a minimum requirement. Considering the design, with a questionnaire sandwiching a single intervention, if the authors wished to maintain their stated aims, there should be slight modification at a minimum. The study aims should be altered to *perceived* changes in confidence and *expected* changes to practice in order to increase validity (Table 2).

**Table 2:** Most significant areas of potential bias identified using CASP checklist

Author	Most significant areas of potential bias identified
Bi et al	• Construct validity bias. As a result of the experimental group also receiving PowerPoint teaching to summarise learning materials it could be argued that the study is examining whether CBL *and* traditional learning methodologies are better than traditional learning methodologies alone. This was not the stated aim of the study • Unclear risk of bias. Methodological information provided lacked in transparency and clarity and thus the extent of other confounding factors is unclear • Instructor bias. Teachers were not blinded and thus any preference or differing expertise in one method may impact on results
Jyothirmayi	• Researcher and confirmation bias. The researcher was involved in conducting 4 of the 6 CBDs and all semi-structured interviews. This may have impacted on behaviour of participants and analysis and interpretation of results in a way that supports the initial hypothesis • Selection bias. It is not clear that selection was random or representative • Small sample size bias. 4 of 6 CBDs were conducted between the primary researcher and a maximum of 3 trainees. This is unlikely representative of broader CBDs across the United Kingdom or Australia
Mackay et al	• Self-selection bias. Trainees across London were invited to participate. Those that attended may have had more favourable pre-existing views on CBL, positively skewing results • Self-reporting and social desirability bias. Participants completed non-anonymised pre and post intervention questionnaires. The relationship between researcher and trainees was unclear. If the researcher was a senior or respected colleague, there is potential for positive skewing of results • Short-term evaluation bias. The short time interval between intervention and subsequent questionnaire may not represent genuine or sustained changes post intervention

## Discussion

To our knowledge, this is the first systematic review to specifically explore evidence for CBL in postgraduate education. Strict inclusion criteria were applied to ensure that the conclusions were applicable to medical oncology trainees. Unfortunately, this review has demonstrated a paucity of data in this area and as such a major limitation of this review was the low number of eligible papers.

Relaxing this study’s inclusion criteria may have increased the generalisability of our conclusions. Several papers were excluded upon full-text review that have relevance to understanding the role of CBL in practicing doctors more broadly. Specifically, three papers were excluded as a result of being deemed not directly related to medical oncology. One of these studies was focussed on haematological malignancies with histopathological review of cases being a prominent feature of learning [19]. The second study focussed on gastrointestinal side effects in cases of childhood leukemia [20, 21]. Note, 43 of the 49 participants in this study were also healthcare professionals other than doctors. The third study was concerned with learning diseases of the oral mucosa in non-cancer patients [22].

,Another reason for study exclusion was lack of participants that were practicing doctors [26–28]. One paper was excluded upon full-text review as 50% of participants were nurses. This study aimed to determine whether the combination of seminars and CBL was superior to traditional LBL in teachings of cancer pain management. It included 30 practicing doctors and concluded that the combination of seminars and CBL was a more effective way of teaching cancer pain management than traditional LBL [23]. Results were not stratified with respect to benefit for doctors and nurses as individual groups, and as such, it is unclear whether the benefit was equal among professions. It is also unclear in this study to what extent benefit was derived as a result of CBL specifically or the teaching in seminars.

It may be beneficial for future reviews to expand inclusion criteria; however, it does appear that there is a paucity of high-quality research in the field of CBL in the medical postgraduate setting [24]. Since 2022, two systematic reviews analysing the quality of RCTs in this field have been published [2, 25]. These studies included resident doctors, occupational health physicians and interns learning about thyroid surgery, management of mental health problems and management of acute dyspnoea, respectively. Conclusions drawn from the three studies included the following: (1) problem-based learning (PBL) appeared more effective than traditional teaching methods in improving performance [26, 27]; (2) PBL was demonstrated to be associated with higher scores for learning motivation, understanding, communication, clinical thinking, self-learning and teamwork skills [26]; and (3) CBL increased knowledge and confidence [28]. Note that the latter’s conclusion was stated as a trend that was not statistically significant, possibly as a result of the small sample size (16) with 56% of participants in the intervention group not attending the planned CBL sessions. The overall quality of data from available RCTs was deemed unsatisfactory [25]. Whilst our systematic review again highlights a lack of robust data, the conclusions of the three studies in this review are consistent with the conclusions of the available studies in other postgraduate specialties as well as in the undergraduate setting.

A strength of this review is that the authors recognised the lack of a universal definition for CBL. A variety of descriptions have been put forward and their features vary [29–33]. Furthermore, there is a significant overlap between a variety of active learning methodologies including PBL and simulated learning. The search strategy (documented in Supplementary Appendix) in this study accounted for this.

Some limitations were identified with the search strategy. Although the initial search appeared broad, identifying 1581 potentially eligible articles, over one third of these were excluded upon title and abstract screen alone as a result of being related to a specific surgical or radiotherapeutic technique. The MeSH terms Radiation Oncology and Surgical Oncology were included in order to ensure no basic cancer science themes were missed. Their addition unfortunately did not yield further eligible studies. One of the three studies included was not identified by the initial literature search, questioning its accuracy and depth [14, 15]. On further review, the article in question used the MeSH term ‘medical students’. Whilst this is technically inaccurate in view of the study’s participants being practicing doctors, it is well recognised that medicine is a career of lifelong learning. As such, there may be similar articles this review failed to identify.

Geographically, CBL may be especially relevant for trainees in rural areas. In New South Wales (NSW), Australia, there are a significant number of trainees living in rural and remote regions [34]. There are seven sites in regional or rural areas which allow for a maximum of 1 accredited trainee [35]. In the era of videoconferencing, a formal CBL programme in oncology speciality training may enhance learning opportunities for regional and remote trainees and increase networking capabilities.

The Chinese study was by far the largest to feature in this review and had the most robust study design. The pathway to speciality oncology training in China however differs significantly from that in Western countries. In Australia, for example, speciality trainees are educated in much smaller cohorts and tend to learn through service delivery. The practice of the Chinese participants appears to more closely resemble that of Australian undergraduate students. It is not clear therefore that the results of this study would be generalisable to speciality trainees in Western countries.

The findings of eligible papers support the notion that practicing doctors value the opportunity for CBL, although no firm conclusions can be drawn regarding postgraduate trainee’s gains in knowledge or performance in view of each study’s limitations. This narrative synthesis highlights the need for further studies in this field. At the current time, the postgraduate education of medical oncology trainees in Australia and New Zealand, under the auspices of the RACP, is based empirically on adequate clinical exposure to the breadth of inpatient and outpatient care undertaken by medical oncologists, adequate supervision by a qualified medical oncologist and sufficient access to academic meetings and resources for self-directed learning [4]. To our knowledge, there is not level 1 evidence for any discrete learning methodology in teaching the practice of medical oncology specifically.

The three studies included in this review hypothesised that CBL would positively impact trainee experience; however, barriers to programme implementation were not assessed. Considering the findings of this review, the authors plan to conduct a feasibility study for a CBL programme within their own institution. Attending trainees will take turns to pick and present an interesting case to their fellow trainees. Through discussion, trainees will identify areas in which they are lacking in knowledge and generate questions. Each trainee will have the following week for self-directed research. Each participant will present a summary of their findings to their colleagues at the following session. Trainees will prospectively document time spent on CBL activities each week and an attendance record will be kept for sessions. Reasons for any trainee absences will be documented. An attendance record of any attending specialists or other attending persons will also be kept. A questionnaire will be completed by all attending trainees after each session assessing perceived utility.

## Conclusion

Eligible studies in this review support the notion that trainees value CBL sessions. This is in keeping with studies from the undergraduate setting where there are indicators that CBL has educational worth and can help develop skills necessary for practicing doctors. The low number of eligible studies and significant methodological flaws in available studies preclude firm conclusions from being drawn in the postgraduate setting. Further research is required to demonstrate whether CBL programmes are feasible within speciality training in Western countries and whether they would have a sustained benefit on trainee confidence and performance.

## Supplementary Information

Below is the link to the electronic supplementary material.Supplementary file1 (DOCX 15 KB)

## Data Availability

The data that support the findings of this study are available from the corresponding author, FD, upon reasonable request.
