# Supplementary material for: Evaluating the Efficacy of Case-Based Learning for Medical Oncology Trainees: A Systematic Review
Source: J Cancer Educ. 2025 Jun 5;41(4):637–45. doi: 10.1007/s13187-025-02651-w (PMC13524996; doi:10.1007/s13187-025-02651-w)
Supplement: Supplementary file 1 [file 13187_2025_2651_MOESM1_ESM.docx]

**Supplementary material**

**Search (Ovid Medline then copied to EMBASE)**
 Embase – 1606, Medline – 375 (total 1981).

 Ovid MEDLINE® including Daily update <1996-current>

1	problem based learning.mp. or Problem-Based Learning/	10850

2	case based learning.mp.	936

3	Simulation Training/ or simulated learning.mp.	6933

4	simulation.mp.	402462

5	flipped classroom.mp.	855

6	collaborative learning.mp. or Interdisciplinary Placement/	1334

7	team based learning.mp.	727

8	1 or 2 or 3 or 4 or 5 or 6 or 7	414579

9	medical oncology.mp. or Medical Oncology/	25824

10	Neoplasms/	351210

11	cancer.mp.	1726424

12	radiation oncology.mp. or Radiation Oncology/	10068

13	surgical oncology.mp. or Surgical Oncology/	4065

14	9 or 10 or 11 or 12 or 13	1828613

15	"Internship and Residency"/ or postgraduate.mp. or Education, Medical/ or Education, Medical, Graduate/	104082

16	physician.mp. or Physicians/	247335

17	"Fellowships and Scholarships"/ or fellows.mp.	14832

18	registrar.mp.	1480

19	Medical Staff/	2179

20	Oncologists/	1801

21	15 or 16 or 17 or 18 or 19 or 20	348145

22	8 and 14 and 21	378
